# Antioxidant, Hypoglycemic, and Hypolipidemic Effects of Puerarin In Vivo

**DOI:** 10.1002/fsn3.70257

**Published:** 2025-05-06

**Authors:** Hong Ruan, Wanqing Li, Huien Chang, Manlin Wen, Shouchun Luo, Fangshuai Song, Li Ye, Jie Mei, Xiqiang Zhu, Xiaopeng Liu, Ning Jiang

**Affiliations:** ^1^ Hubei Key Laboratory of Biologic Resources Protection and Utilization Hubei Minzu University Enshi People's Republic of China; ^2^ School of Biological Science and Technology Hubei Minzu University Enshi People's Republic of China

**Keywords:** aging, antioxidant, hypoglycemic, hypolipidemic, oxidative stress, puerarin

## Abstract

Puerarin (PUE) exhibits various pharmacological effects. This study evaluated its antioxidant, hypoglycemic, and hypolipidemic effects in vivo using models of aging, diabetes, and hyperlipidemia. D‐galactose‐induced aging, streptozotocin (STZ)‐induced diabetic, and high‐fat diet‐induced hyperlipidemic mouse models were established. To evaluate the therapeutic effects, mice were administered various doses of PUE (50, 100, and 200 mg/kg). Results showed that PUE treatment improved antioxidant enzyme activities and reduced serum and liver malondialdehyde (MDA) levels in aging mice, thereby mitigating cellular oxidative stress. In diabetic mice, fasting blood glucose (FBG) levels were observed to decrease, while hepatic hexokinase (HK) activity, pyruvate kinase (PK) activity, and insulin levels increased after 7 weeks of PUE treatment. Furthermore, PUE significantly enhanced blood lipid profiles and antioxidant enzyme properties in diabetic mice. In hyperlipidemic mice, PUE administration led to decreased levels of total cholesterol (TC), triglycerides (TG), and low‐density lipoprotein cholesterol (LDL‐C), while increasing high‐density lipoprotein cholesterol (HDL‐C). These findings indicate that PUE possesses antioxidant, hypoglycemic, and hypolipidemic properties and shows potential for treating aging and related diseases like type 2 diabetes and hyperlipidemia.

## Graphical abstract

The antioxidant, hypoglycemic, and hypolipidemic effects of puerarin in vivo were investigated. The results demonstrated that it reduces oxidative stress in senescent mice, significantly improves blood lipid levels and antioxidant enzyme activity in diabetic mice, and supports normal pancreatic function. This study provides some useful information for the development and utilization of puerarin.

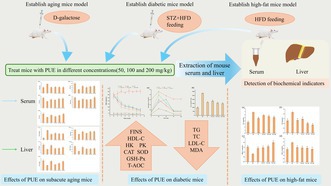

## 1 Introduction

According to the United Nations World Population Prospects 2024, the proportion of people aged 65 and over will reach 17% globally by 2024, with projections suggesting it will rise to 33% by 2054 and exceed 40% by 2100 (United Nations Department of Economic and Social Affairs 2024). Aging is an important high‐risk element for a variety of metabolic diseases. It is an irreversible physiological process where the functions of cells, tissues, and organs gradually decline due to genetic, environmental, and lifestyle factors (Koltover 2017). Aging mechanisms are complex, with theories including free radical theory, mitochondrial damage theory, cellular autophagy theory, and telomere theory (Doan et al. 2015; Ma 2014). Free radical theory, in particular, has been extensively studied, suggesting that low intracellular concentrations of reactive oxygen species (ROS) are essential for cell responses to signaling, homeostasis, and stress (Kim et al. 2024). With age, the balance of free radicals in cells is disrupted, leading to excessive ROS production and increased oxidative stress (Lichtenberg and Pinchuk 2015). This oxidative stress triggers inflammatory responses, accelerates DNA damage, and induces cellular senescence (Chen et al. 2023), contributing to organ damage and aging‐related diseases. Type 2 diabetes mellitus (T2DM) is closely associated with aging (Gunasekaran and Gannon 2011; Leahy et al. 2015) and leads to abnormalities in glucose‐lipid metabolism and hepatic insulin pathways. Research indicates that oxidative stress during aging promotes cellular senescence and the release of aging‐related secretory factors, which mediate pancreatic cell dysfunction, adipose tissue dysfunction, and insulin resistance, thus contributing to T2DM (Takaaki et al. 2022). Furthermore, oxidative stress is an essential factor leading to hyperlipidemia as well as its complications. It disrupts lipid metabolism, leading to excessive lipid accumulation and blood transport disorders in the elderly, resulting in hyperlipidemia (Song et al. 2023). Long‐term use of drugs to manage aging and related diseases can lead to side reactions and potentially worsen the condition (Chovsepian et al. 2023; Pan et al. 2022). Exploration of new therapies to alleviate age‐related metabolic diseases and improve the health of the elderly is therefore urgently needed.

Puerarin (PUE), a flavonoid derived from the dried rhizome of *Pueraria lobata* (Willd.) Ohwi, is known for its potential health benefits. Pharmacological studies have confirmed that PUE possesses antioxidant (Jeon et al. 2020), hypoglycemic effects (Liu et al. 2021), anti‐inflammatory (Ding et al. 2023), and hypolipidemic (Xu et al. 2021a). These properties make it a candidate for treating various aging‐related diseases, such as diabetes mellitus (Zhang et al. 2022b), sleep disorders in the elderly (Tao et al. 2023), osteoporosis (Cao et al. 2022), and cerebrovascular disease (Lv et al. 2022).

Research has suggested that PUE exhibits strong antioxidant activity by regulating the nuclear factor erythroid 2‐related factor 2 (Nrf2) pathway and the expression of antioxidant enzymes (Jeon et al. 2020). Additionally, PUE extends the lifespan of *Drosophila* by modulating target of rapamycin (TOR) signaling‐mediated lysosomal autophagy (Kang et al. 2023). PUE also reduces inflammation and dyslipidemia in obese mice through regulation of macrophages and tumor necrosis factor‐α (TNF‐α) (Noh et al. 2022). Additionally, it acts on the pancreas, the liver, the skeletal muscles, and the adipose tissue to produce hypoglycemic effects through the restoration of their physiological functions (Bai et al. 2022). Given these findings, PUE may offer promising therapeutic effects for aging and related metabolic diseases.

Regarding anti‐aging effects, PUE has been shown to have beneficial effects in *Drosophila* (Kang et al. 2023), but its impact on mice has not yet been studied. In terms of hypoglycemia, PUE has been proved to reduce FBG levels inT2DM, increase serum insulin levels, and alleviate oxidative stress damage to pancreatic tissue function and mitochondrial structure (Liang et al. 2022; Xu et al. 2016). However, more in‐depth studies are needed. For hypolipidemic effects, research has examined the impact of high concentrations of PUE on blood lipid levels in hyperlipidemic mice, but further investigation is required to determine whether these effects are dose‐dependent (Noh et al. 2022).

This study was designed to explore whether PUE has therapeutic effects on aging‐related diseases by investigating its antioxidant, hypoglycemic, and hypolipidemic effects at different concentrations. The research will be conducted using D‐galactose‐induced aging model mice, diabetic model mice, and high‐fat diet‐induced model mice. The findings will lay the foundation for further development of PUE as a health food or anti‐aging medicine.

## 2 Materials and Methods

### 2.1 Laboratory Animals and Feed

Six‐week‐old male C57BL/6 mice (20 ± 2 g) and five‐week‐old male Kunming mice (20 ± 2 g) were provided by the Laboratory Animal Center of Three Gorges University (Hubei, China). The laboratory animal qualification certificate is SYXK(E)2016‐0057.

#### 2.1.1 High‐Fat Feed

The high‐fat feed included irradiated experimental animal pellet feed with the following composition: 0.2% sodium cholate, 15% sugar, 1% cholesterol, 5% egg yolk powder, 10% lard, and 68.8% basic feed. This feed was obtained from Wuhan Wuchang District Chunzhilong Experimental Animal Feed Co. (Wuhan, China).

#### 2.1.2 Conventional Feed

The conventional feed consisted of 60% cereal energy (corn, wheat, alfalfa grass, and broken rice), 34% protein (fish meal, chicken meal, and soybean meal), and 4% amino acids, vitamins, and minerals. This feed was provided by Jiangsu Synergy Pharmaceutical Biological Co. (Jiangsu, China).

### 2.2 Materials and Reagents

Puerarin was provided by Xian Tengyun Biotechnology Co. Ltd. (Xian, China). STZ, metformin hydrochloride, D‐galactose, and Vitamin C were provided by Sinopharm Chemical Reagent Co. (Shanghai, China). Lovastatin was sourced from Shanghai Jiechun Biotechnology Co. Ltd. (Shanghai, China). Total cholesterol (TC), high‐density lipoprotein (HDL‐C), low‐density lipoprotein (LDL‐C), malondialdehyde (MDA), triglycerides (TG), glutathione peroxidase (GSH‐Px), total antioxidant capacity (T‐AOC), superoxide dismutase (SOD), catalase (CAT), total protein quantification, hexokinase (HK) and pyruvate kinase (PK) test kits, as well as mouse insulin (INS) ELISA test kits were acquired from Nanjing Jiancheng Bioengineering Institute (Nanjing, China). All experimental reagents are analytical pure.

### 2.3 Methods

#### 2.3.1 Breeding of Experimental Animals

Sixty 6‐week‐old male C57BL/6 mice and 120 5‐week‐old male Kunming mice, each weighing 20 ± 2 g, were housed at Hubei Minzu University (temperature 23°C ± 1°C, humidity 60%, 12 h light/dark cycle). All mice were acclimated with a normal diet for 5 days. All animal procedures adhered to local regulations and ethical guidelines for the care and use of laboratory animals.

#### 2.3.2 Mouse Serum and Liver Homogenates Prepared

After the treatment period, mice were fasted for 8 h (without water). Blood was collected by removing the eyeballs and centrifuged at 3500 rpm for 15 min. The serum was collected and stored at −20°C. The liver was removed, washed with saline, weighed, and cut into pieces. Nine mL of PBS buffer per gram of liver tissue was added for homogenization, followed by centrifugation at 2500 rpm for 10 min. This supernatant was stored at −80°C.

#### 2.3.3 Effects of PUE on Subacute Aging Mice

##### 2.3.3.1 Grouping, Modeling, and Administration of Experimental Animals

Sixty 5‐week‐old male Kunming mice were randomly divided into six groups, each containing 10 mice: including aging blank control group (BC), aging model control group (MC), aging positive control group (PC), PUE low‐dose group (PUE‐50), PUE medium‐dose group (PUE‐100), and PUE high‐dose group (PUE‐200). Based on the study by Zhang et al. (2020a), a model was established, and the positive administration dose was determined as follows: the BC group was injected with an equal volume of saline, while the other groups were subcutaneously injected with 200 mg/kg of D‐galactose daily. Concurrently, treatments were administered by gavage: The PUE‐50, PUE‐100, and PUE‐200 groups were given 50, 100, and 200 mg/kg of PUE, respectively, and the dose of PUE was determined based on previous experiments (same as below) (Gao et al. 2021). The PC group received 200 mg/kg of Vitamin C (Vc) daily. The BC and MC groups received an equal volume of blank solution. This regimen continued for a total of 8 weeks.

##### 2.3.3.2 Determination of Antioxidant Activity In Vivo

The activities of MDA, GSH‐Px, CAT, SOD, and T‐AOC in mouse serum and liver tissue were measured with the corresponding assay kits.

#### 2.3.4 Effects of PUE on Diabetic Mice

##### 2.3.4.1 Grouping, Modeling, and Administration of Experimental Animals

Sixty 6‐week‐old male C57BL/6 mice were randomly divided into six groups, each consisting of 10 mice: including diabetic blank control group (BC), diabetic model control group (MC), diabetic positive control group (PC), PUE low‐dose group (PUE‐50), PUE medium‐dose group (PUE‐100), and PUE high‐dose group (PUE‐200). A type 2 diabetes mellitus (T2DM) model was induced with modifications based on Zhang et al. (2022a, ). All groups, except the BC group, were intraperitoneally injected with 50 mg/kg of STZ solution for 4 days. The BC group received an equal volume of citrate buffer. Coat color, mental condition, and urination of the mice were observed daily. The fasting blood glucose level was determined 3 days after the last STZ injection. Mice with fasting blood glucose levels ≥ 11.1 mmol/L were considered to beT2DM model mice. Following successful modeling, distilled water was administered by gavage to the BC and MC groups, the PC group was gavaged with 200 mg/kg of metformin hydrochloride, and the PUE groups individually received 50, 100, and 200 mg/kg of PUE. Treatments were administered daily for 7 weeks. From the start of STZ injections, all groups were fed a high‐fat diet excluding the BC group, which was fed conventional feed.

##### 2.3.4.2 Measurement of Fasting Blood Glucose (FBG) Concentration

Blood samples of the orbital venous plexus were taken weekly to determine blood glucose levels. The serum is collected according to the method described in Section 2.3.2, and the glucose level is determined with a glucose measurement kit.

##### 2.3.4.3 Oral Glucose Tolerance Test (OGTT)

After the treatment period, mice were fasted for 8 h before performing the OGTT. All groups were gavaged with a 2 g/kg glucose solution. Blood samples of the orbital venous plexus were collected at 0, 0.5, 1, 1.5, and 2 h after gavage to measure blood glucose concentrations. These measurements were used to construct a blood glucose curve over time, and the area under the curve (AUC) was calculated (Xu et al. 2021b).

##### 2.3.4.4 Fasting Serum Insulin (FINS) Detection

Following completion of the treatment, blood samples were centrifuged at 3500 rpm for 15 min to obtain serum. Serum insulin levels were measured using an ELISA assay.

##### 2.3.4.5 Detection of Blood Lipid Levels

Total cholesterol (TC), TG, HDL‐C, and LDL‐C levels in serum were determined individually with the respective assay kits.

##### 2.3.4.6 Determination of Liver Hexokinase (HK) and Pyruvate Kinase (PK) Activities

The activities of hexokinase (HK) and pyruvate kinase (PK) in liver homogenates were measured following the instructions provided with the respective assay kits.

##### 2.3.4.7 Detection of Antioxidant Levels

Serum and liver levels of GSH‐Px, SOD, CAT, T‐AOC, and MDA of mice were measured with the corresponding assay kits.

#### 2.3.5 Effects of PUE on High‐Fat Mice

##### 2.3.5.1 Grouping, Modeling, and Administration of Experimental Animals

Sixty 5‐week‐old male Kunming mice were randomly divided into six groups, with 10 mice in each group: including high‐fat blank control (BC), high‐fat model control (MC), high‐fat positive control (PC), PUE low‐dose (PUE‐50), PUE medium‐dose (PUE‐100), and PUE high‐dose (PUE‐200). As described by Li et al. (2023), the BC group was fed a conventional diet, while the MC, PC, and PUE groups were fed a high‐fat diet for modeling purposes. The PC group was administered 3 mg/kg of lovastatin per day by oral gavage, and 50, 100, and 200 mg/kg of PUE were administered daily by gavage to the PUE groups, respectively. Both the BC group and the high‐fat model group received an equal volume of blank solution by gavage for 8 weeks of continuous treatment.

##### 2.3.5.2 Detection of Blood Lipid Levels in High‐Fat Model Mice

Serum levels of TC, TG, HDL‐C, and LDL‐C in the high‐fat model mice were measured according to the kit instructions.

### 2.4 Statistical Analysis

Data were described as mean ± standard error (mean ± SE) and analyzed using SPSS 20.0. The Kolmogorov–Smirnov test was used to assess data normality, and the Levene test was applied for homogeneity of variance. Differences between two groups were assessed using a Student's *t*‐test (for normally distributed data), with Welch correction for unequal variances. For non‐normally distributed data, the non‐parametric Kruskal–Wallis *H* test was used. A *p* value < 0.05 was considered significant, and *p* < 0.01 was considered extremely significant. Figures were created using Origin 2018.

## 3 Results

### 3.1 Effects of PUE on Subacute Senescence Mice

**FIGURE 1:**
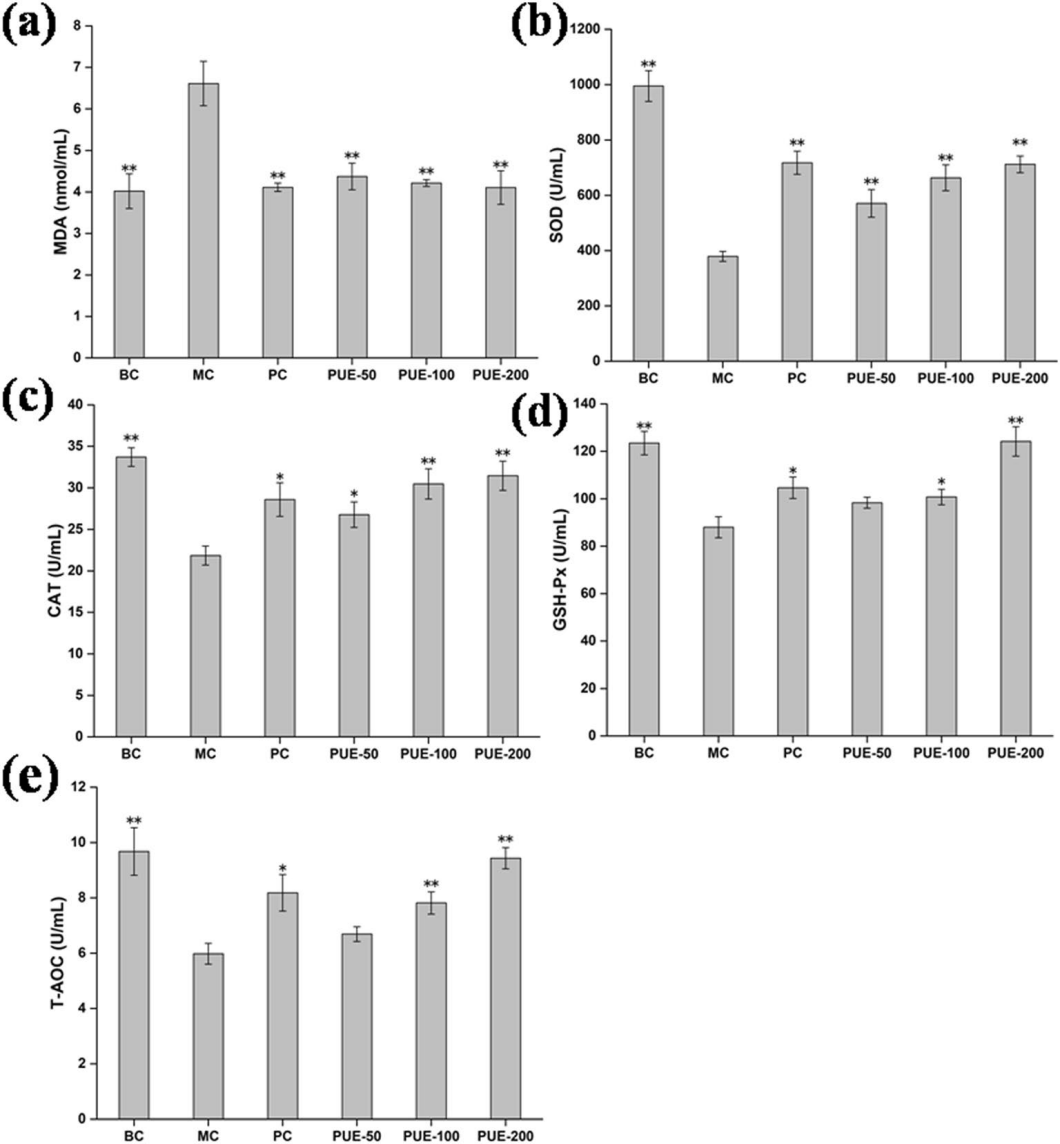
Effects of PUE on oxidative indices in the serum of subacute aging mice (*n* = 10, $\bar{x}\pm \mathrm{SE}$). (a) MDA; (b) SOD; (c) CAT; (d) GSH‐Px; (e) T‐AOC. * and ** indicate that *p* < 0.05 and *p* < 0.01, respectively, compared to the MC group.

**FIGURE 2:**
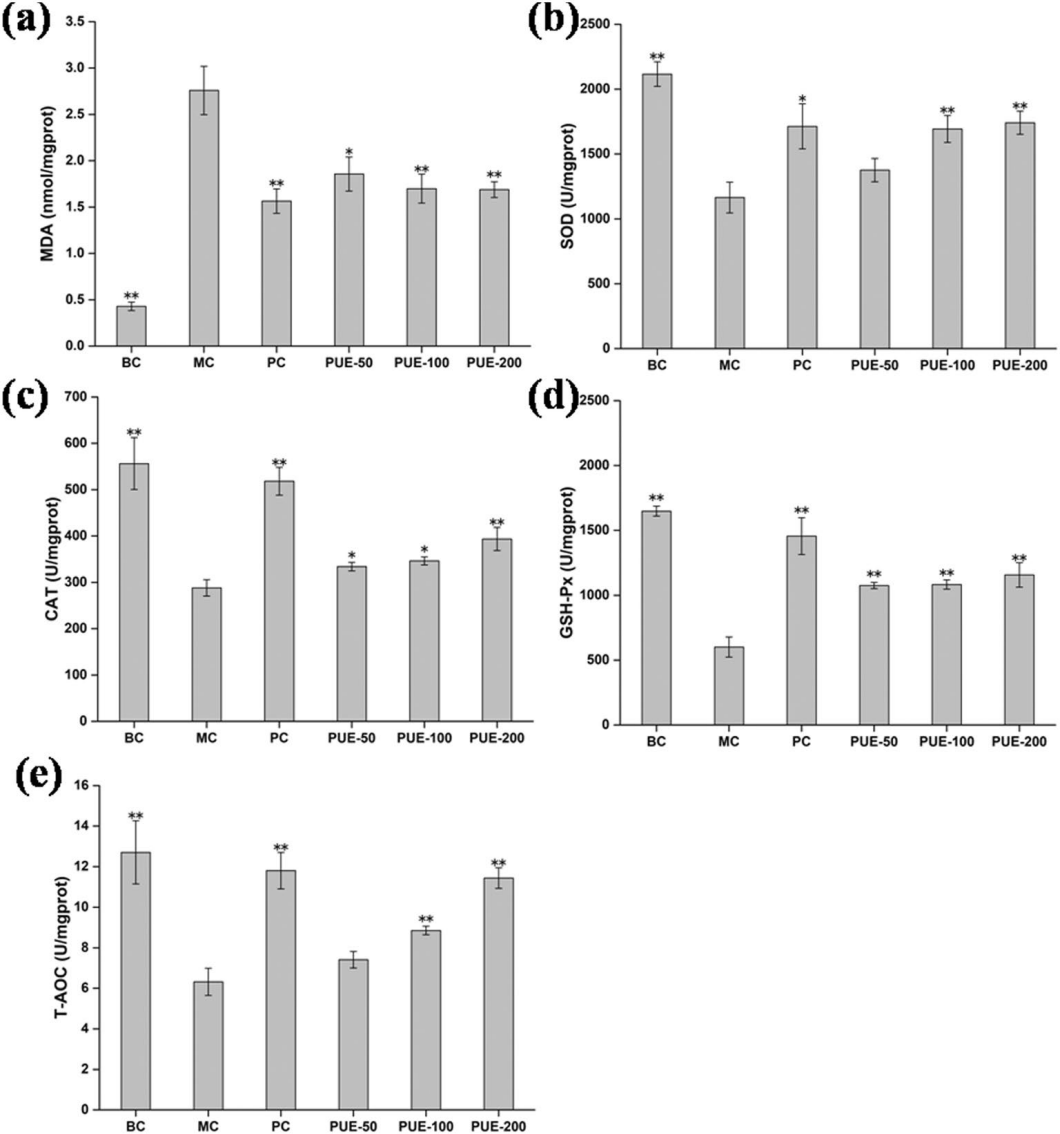
Effects of PUE on oxidative indices in the liver of subacute aging mice (*n* = 10, $\bar{x}\pm \mathrm{SE}$). (a) MDA; (b) SOD; (c) CAT; (d) GSH‐Px; (e) T‐AOC. * and ** indicate that *p* < 0.05 and *p* < 0.01, respectively, versus the MC group.

Serum and liver MDA levels of senescence model mice were significantly higher than healthy mice in the BC group (*p* < 0.01). Conversely, the levels of SOD, CAT, GSH‐Px, and T‐AOC in the senescence model mice were remarkably lower in comparison to the BC group (*p* < 0.01). Following administration of various concentrations of PUE, serum and liver MDA levels were remarkably decreased in the PUE groups versus the MC group (*p* < 0.05), with a dose‐dependent effect observed. The MDA reduction in the PUE‐200 group was comparable to that achieved with Vc. Additionally, SOD, CAT, GSH‐Px, and T‐AOC levels in the serum and liver of mice in the PUE groups increased to varying extents (Figures 1 and 2).

### 3.2 Effects of PUE on Diabetic Mice

#### 3.2.1 Effect of PUE on FBG in Diabetic Mice

**FIGURE 3:**
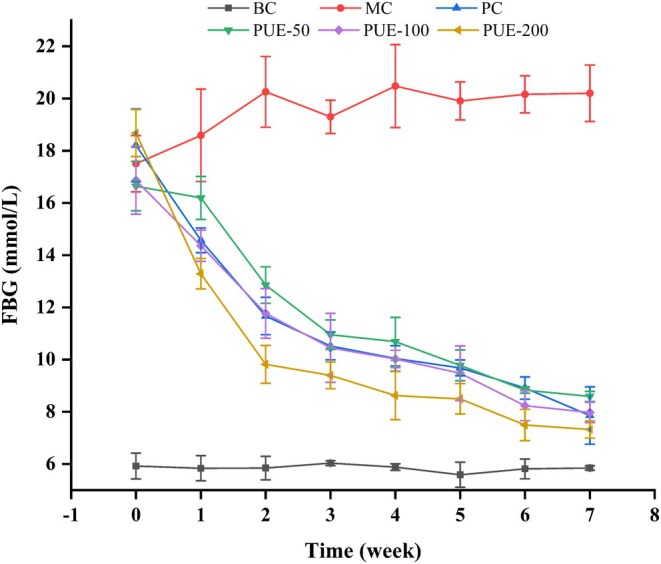
Effect of PUE on FBG concentration in diabetic mice (*n* = 10, $\bar{x}\pm \mathrm{SE}$).

During the test period, fasting blood glucose (FBG) levels maintained relatively consistency in the BC group, while FBG levels in healthy mice increased sharply following STZ injection, as shown in Figure 3. With PUE intervention, FBG levels began to decrease in the first week, stabilized after 4 weeks, and in the PUE groups, the FBG levels were comparable to that of the PC group after 7 weeks.

#### 3.2.2 Effect of PUE on OGTT in Diabetic Mice

**FIGURE 4:**
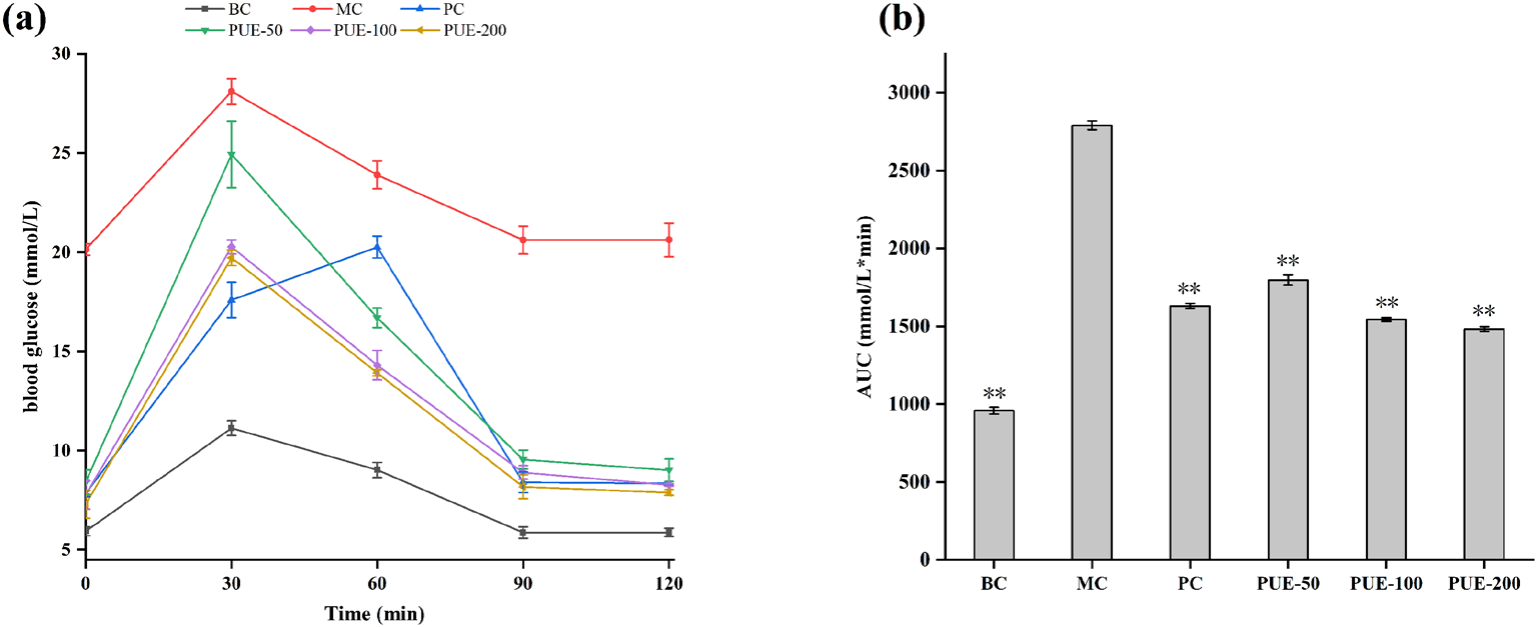
Results of OGTT on diabetic mice (*n* = 10, $\bar{x}\pm \mathrm{SE}$). (a) oral glucose tolerance test; (b) area under curve. * and ** indicate that *p* < 0.05 and *p* < 0.01, respectively.

Following the completion of the treatment, the OGTT was conducted to assess whether PUE could improve glucose metabolism in diabetic mice. As shown in Figure 4, blood glucose levels reached a peak at 30 min and then declined in all groups. In the PUE groups, this decrease was dose‐dependent, with levels nearly returning to baseline after 120 min. Glucose tolerance was improved in both the PUE and PC groups, with lower blood glucose levels at all time points compared to the MC group. By analyzing the AUC, a significant (*p* < 0.01) and dose‐dependent reduction in AUC was observed in the PUE‐50, PUE‐100, and PUE‐200 groups in comparison to the MC group, with the PUE‐100 and PUE‐200 groups showing results comparable to the PC group.

#### 3.2.3 Fasting Insulin Levels

**FIGURE 5:**
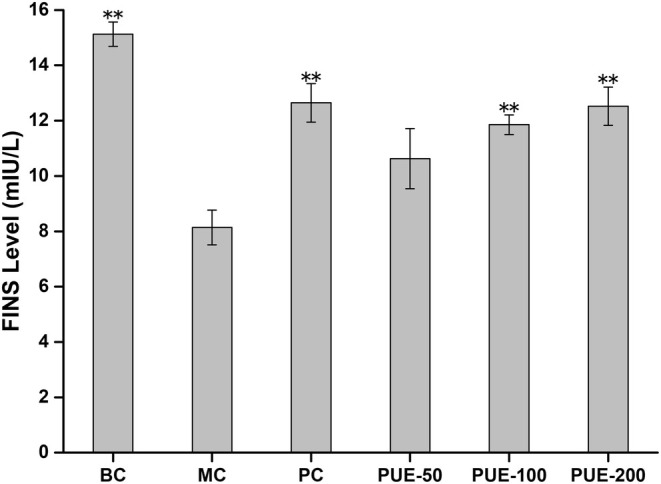
Effect of PUE on fasting insulin level in diabetic mice (*n* = 10, $\bar{x}\pm \mathrm{SE}$). * and ** indicate that *p* < 0.05 and *p* < 0.01, respectively, compared to the MC group.

The FINS levels were significantly reduced in diabetic mice induced by STZ (*p* < 0.01), as shown in Figure 5. Following the intervention, there was a notable elevation in FINS levels in the PUE‐100 and PUE‐200 groups, as well as in the PC group, versus the MC group (*p* < 0.01 for the PUE‐100 and PUE‐200 groups; *p* < 0.01 for the PC group).

#### 3.2.4 Lipid Levels in Diabetic Mice

Following an intraperitoneal injection of STZ, the concentrations of TC, TG, and LDL‐C were markedly increased (*p* < 0.01), while the concentration of HDL‐C was significantly decreased (*p* < 0.01), indicating abnormal lipid levels in the mice. After administration of PUE, TG, TC (except in the PUE‐50 group), and LDL‐C concentrations were significantly reduced (*p* < 0.01), and HDL‐C concentration was significantly elevated (*p* < 0.01) in the PUE‐50, PUE‐100, and PUE‐200 groups. The PUE‐200group showed lipid level changes comparable to those observed with metformin hydrochloride, which effectively decreased TG, TC, and LDL‐C and increased HDL‐C (Table 1).

**TABLE 1:** Effect of PUE on blood lipid levels in diabetic mice (*n* = 10, $\bar{x}\pm \mathrm{SE}$).

Group	BC	MC	PC	PUE‐50	PUE‐100	PUE‐200
TG (mmol/L)	0.66 ± 0.02**	1.28 ± 0.11	0.78 ± 0.06**	0.92 ± 0.05*	0.82 ± 0.05**	0.78 ± 0.07**
TC (mmol/L)	2.61 ± 0.22**	4.80 ± 0.24	3.58 ± 0.14**	4.32 ± 0.17	3.63 ± 0.30**	3.54 ± 0.21**
LDL‐C (mmol/L)	0.81 ± 0.06**	1.98 ± 0.18	1.08 ± 0.07**	1.17 ± 0.06**	0.96 ± 0.07**	0.95 ± 0.06**
HDL‐C (mmol/L)	1.96 ± 0.15**	0.80 ± 0.11	2.60 ± 0.15**	1.82 ± 0.07**	2.18 ± 0.20**	2.35 ± 0.13**

*Note:* * and ** indicate that *p* < 0.05 and *p* < 0.01, respectively, versus the MC group.

#### 3.2.5 Liver HK and PK Levels

**FIGURE 6:**
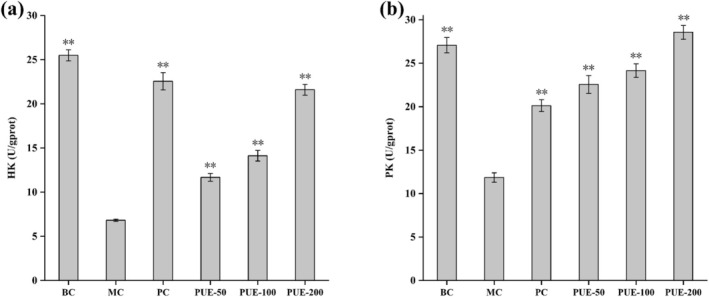
Effect of PUE on HK and PK in diabetic mice (*n* = 10, $\bar{x}\pm \mathrm{SE}$). (a) HK; (b) PK. * and ** indicate that *p* < 0.05 and *p* < 0.01, respectively, versus the MC group.

This can be seen by observing Figure 6, both pyruvate kinase (PK) and hexokinase (HK) levels were significantly reduced in the MC group (*p* < 0.01), compared to the BC group. Following treatment with metformin and PUE, HK, and PK levels approached or even returned to normal.

#### 3.2.6 Effect of PUE on Antioxidant Activity in Diabetic Mice

The MC group had significantly higher MDA levels in serum and liver than the BC group (*p* < 0.01) as shown in Table 2. Treatment with PUE resulted in a significant (*p* < 0.05), dose‐dependent reduction in MDA levels. The diabetic mice in the PUE group had significantly higher levels of CAT, SOD, GSH‐Px, and T‐AOC in their serum (*p* < 0.01). Notably, the levels of GSH‐Px and T‐AOC were even higher in the PUE‐200 group than in the BC group. All PUE doses, except for the PUE‐50, led to a significant increase in liver T‐AOC activity (*p* < 0.01). Additionally, all PUE doses significantly enhanced CAT, SOD, and GSH‐Px activities in their liver (*p* < 0.01). Specifically, low‐, medium‐, and high‐doses of PUE increased serum T‐AOC by 44%, 176%, and 233%, and liver T‐AOC by 10%, 30%, and 60%, respectively, versus the MC group.

**TABLE 2:** Effect of PUE on antioxidant indexes of diabetic mice (*n* = 10, $\bar{x}\pm \mathrm{SE}$).

	Group	BC	MC	PC	PUE‐50	PUE‐100	PUE‐200
Serum	MDA (nmol/mL)	2.81 ± 0.41**	9.57 ± 0.51	4.86 ± 0.33**	5.11 ± 0.30**	5.05 ± 0.27**	4.65 ± 0.20**
	CAT (U/mL)	553.78 ± 22.24**	300.21 ± 20.99	536.02 ± 5.53**	409.62 ± 7.35**	455.34 ± 9.92**	495.37 ± 24.50**
	SOD (U/mL)	324.30 ± 12.13**	80.01 ± 5.02	233.14 ± 10.87**	154.02 ± 4.10**	159.00 ± 3.59**	177.56 ± 10.38**
	GSH‐Px (U/mL)	1272.15 ± 41.87**	877.93 ± 76.75	1363.73 ± 23.78**	1222.20 ± 56.74**	1396.20 ± 31.42**	1401.70 ± 16.40**
	T‐AOC (U/mL)	3.48 ± 0.27**	1.06 ± 0.05	2.62 ± 0.12**	1.52 ± 0.12**	2.91 ± 0.11**	3.51 ± 0.08**
Liver	MDA (nmol/mgprot)	0.41 ± 0.05**	2.58 ± 0.25	1.81 ± 0.04*	1.95 ± 0.07*	1.66 ± 0.13**	1.33 ± 0.04**
	CAT (U/mgprot)	100.83 ± 4.80**	47.73 ± 3.56	68.35 ± 2.56**	74.07 ± 2.81**	94.85 ± 1.68**	111.01 ± 6.45**
	SOD (U/mgprot)	25.33 ± 0.75**	13.17 ± 0.07	21.50 ± 0.55**	19.25 ± 0.38**	22.56 ± 0.53**	26.59 ± 0.58**
	GSH‐Px (U/mgprot)	1046.70 ± 31.70**	697.89 ± 14.75	969.95 ± 28.85**	865.50 ± 20.86**	1149.48 ± 38.63**	1254.65 ± 32.65**
	T‐AOC (U/mgprot)	3.57 ± 0.09**	2.35 ± 0.11	3.16 ± 0.08**	2.60 ± 0.07	3.06 ± 0.07**	3.76 ± 0.06**

*Note:* * and ** indicate that *p* < 0.05 and *p* < 0.01, respectively, versus the MC group.

### 3.3 Effects of PUE on High‐Fat Mice

**FIGURE 7:**
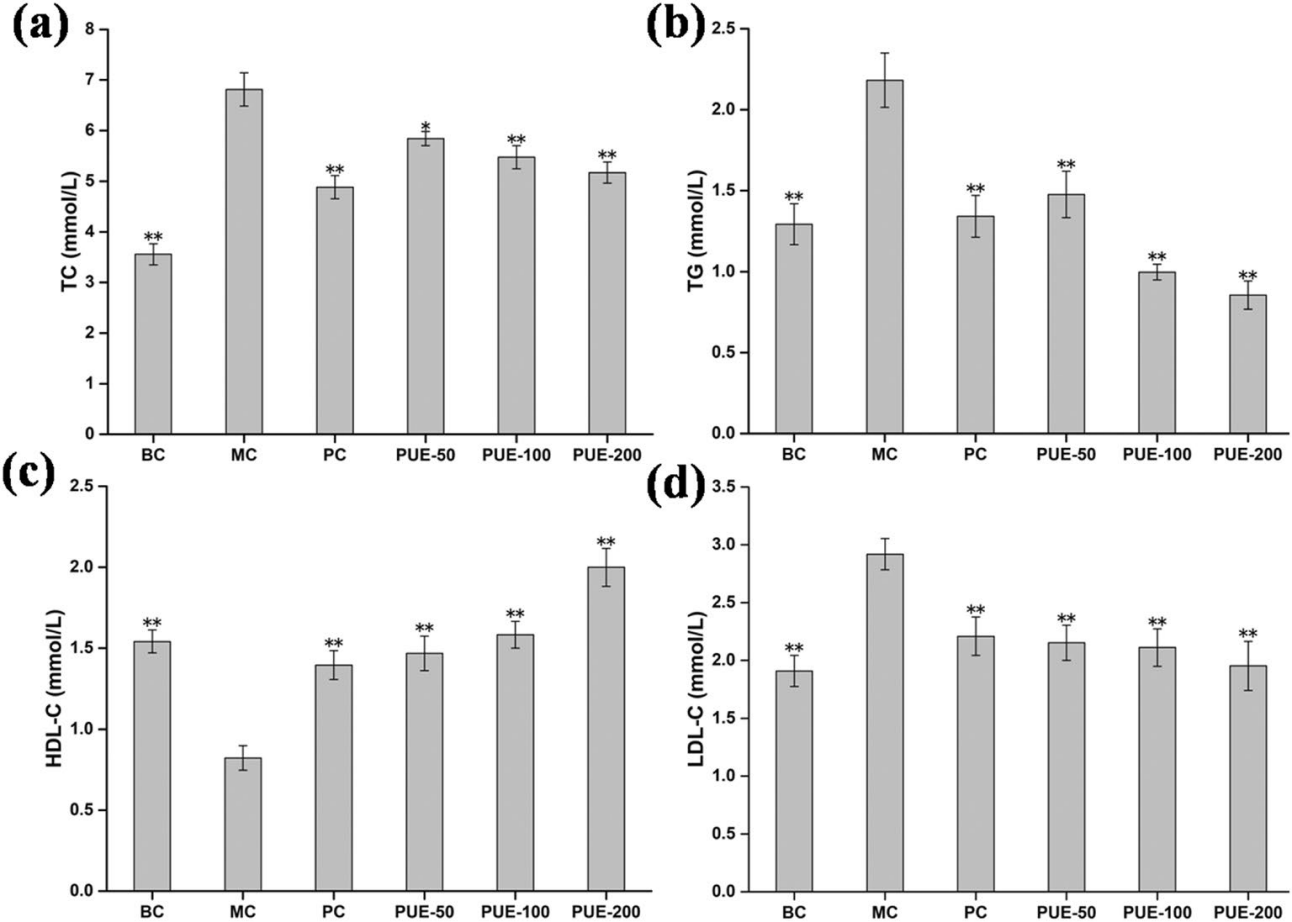
Effects of PUE on blood lipid levels in hyperlipidemia mice (*n* = 10, $\bar{x}\pm \mathrm{SE}$). (a) TC; (b) TG; (c) HDL‐C; (d) LDL‐C. * and ** indicate that *p* < 0.05 and *p* < 0.01, respectively, versus the MC group.

As illustrated in Figure 7, the contents of TC, TG, and LDL‐C in the serum of mice in the high‐fat model group were significantly elevated (*p* < 0.01) compared to the BC group, providing confirmation of the successful establishment of the hyperlipidemic mouse model. The levels of TC (except for PUE‐50 group), TG, and LDL‐C in the PUE and PC groups were significantly reduced following drug intervention in comparison to the MC group (*p* < 0.01), while HDL‐C levels were markedly elevated (*p* < 0.01). The therapeutic effects of low‐, medium‐, and high‐dose PUE were dose‐dependent, leading to a noticeable alleviation of pathological symptoms in the mice, particularly in the PUE‐200 group.

## 4 Discussion

Increased intracellular inflammation and immune responses are major contributors to age‐related diseases, which are highly correlated with elevated oxidative stress. Oxidative stress triggers the expression of nuclear factor kappa‐light‐chain‐enhancer of activated B cells (NF‐κB), interleukin‐6 (IL‐6), tumor necrosis factor‐alpha (TNF‐α), cyclooxygenase‐2 (COX‐2), and inducible nitric oxide synthase, leading to inflammatory responses (Chung et al. 2006), which in turn contribute to aging and the occurrence of metabolic disorders such as type 2 diabetes and hyperlipidemia (Inoue et al. 2018). Additionally, oxidative stress impairs mitochondrial function and energy metabolism, which are linked to the development of type 2 diabetes mellitus (Chen et al. 2023). Emerging evidence suggests that plant flavonoids, including baicalin (Li et al. 2019), kaempferol (Alshehri 2021), and isosinensetin (Qin et al. 2023), offer therapeutic benefits for aging‐related diseases. Our study found that PUE exhibited positive effects in treating senescent, diabetic, and hyperlipidemic mice, suggesting the potential of PUE to treat age‐related diseases.

Long‐term continuous injection of D‐galactose leads to an elevation in intracellular reactive oxygen species (ROS) levels, which in turn reduces antioxidant enzymes (Dehghani et al. 2019). This disruption of the antioxidant defense system exacerbates lipid peroxidation, increases lipofuscin, and causes organ dysfunction, resulting in physiological and biochemical changes similar to natural aging, making it a widely used model in anti‐aging drug studies (Zhang et al. 2020b). In healthy mice injected with 200 mg/kg D‐galactose, serum and liver MDA levels increased, leading to oxidative stress and hepatocyte injury, consistent with the finding of Zhang et al. (2020a, ). SOD, CAT, and GSH‐Px are significant antioxidant enzymes for scavenging free radicals as well as reducing ROS levels. Long‐term D‐galactose injection caused excessive accumulation of hydrogen peroxide (H_2_O_2_) and galactitol, inhibiting the Nrf2 pathway and significantly reducing serum and liver levels of SOD, CAT, and GSH‐Px, thereby decreasing T‐AOC. The Nrf2/ARE signaling pathway is known to protect cells from oxidative damage. PUE markedly increases Nrf2 gene expression and protein levels in cells from mice with colitis, inducing antioxidant enzymes (SOD, CAT, etc.) and alleviating oxidative stress damage (Jeon et al. 2020). The Nrf2/ARE signaling pathway is known to protect cells from oxidative damage. PUE significantly increases Nrf2 gene expression and protein levels in cells from mice with colitis, inducing antioxidant enzyme production (SOD, CAT, etc.) and alleviating oxidative stress damage (Jeon et al. 2020). Administering PUE to subacute aging model mice resulted in an increase in SOD, GSH‐Px, CAT, and T‐AOC levels, and significantly reduced MDA levels in serum and liver. PUE likely activates the Nrf2/ARE pathway by upregulating Nrf2 expression and promotes the transcription and translation of antioxidant enzyme genes. Additionally, the MAPK signaling pathway, an upstream regulator of the Nrf2, is also implicated. Mo et al. (2022) indicated that PUE inhibits the overexpression of extracellular signal‐regulated kinase (ERK), c‐jun amino‐terminal kinase (JNK)and p38, suggesting that PUE may reduce oxidative stress damage in aging mice by regulating the MAPK signaling pathway, highlighting its antioxidant potential for the protection against age‐related diseases.

Aging is a significant factor causing type 2 diabetes mellitus (T2DM). Pancreatic cell senescence reduces β‐cell gene expression, while senescent cells express a senescence‐associated secretory phenotype (SASP) (Midha et al. 2021). This phenotype influences adipose tissue and pancreatic β‐cell function by releasing pro‐inflammatory cytokines and chemokines, which affects insulin release and increases the susceptibility of older adults to T2DM (Aguayo‐Mazzucato et al. 2019). STZ, a DNA alkylation agent, induces oxidative stress and alkylation damage to pancreatic islet cells and influences the kidneys, liver, and adipose tissue functions, commonly used to induce type 2 diabetes (Gromotowicz‐Poplawska et al. 2019; Kim et al. 2016). A diabetes model was induced in mice by administering STZ (50 mg/kg) intraperitoneally for 4 consecutive days. This induction method aligns with the study by Zhang et al. (2022a, ), confirming its validity. Results showed a significant elevation in FBG and AUC levels, a reduction in fasting insulin (FINS) levels, and a marked reduction in hexokinase (HK) and pyruvate kinase (PK) levels, indicating elevated blood glucose, reduced insulin sensitivity, and impaired glucose metabolism in diabetic mice. Following PUE intervention in STZ‐induced diabetic mice, the FBG and AUC values were significantly reduced in the treated group compared to the control, while FINS, HK, and PK levels significantly increased, suggesting that PUE was effective in reducing blood glucose and improving glucose metabolism in diabetic mice. Previous research suggests that PUE mainly lowers blood glucose by modulating multiple signaling pathways in the pancreas, adipocytes, skeletal muscle, and liver. It upregulates glucagon‐like peptide‐1 receptor (GLP‐1R) expression in the pancreas (Wang et al. 2020), inhibits caspase family proteins and AIF (Liang et al. 2019), promoting β‐cell proliferation, preventing β‐cell apoptosis, increasing serum insulin levels, alleviating insulin resistance, and reducing fasting blood glucose. Additionally, elevated fasting blood glucose in type 2 diabetes is associated with hepatic gluconeogenesis. PUE activates PI3K/Akt signaling and phosphorylates fork head box protein o1 (FOXO1), which reduces the expression of glucose 6‐phosphatase (G6Pase) and phosphoenolpyruvate carboxy kinase (PEPCK), inhibiting hepatic gluconeogenesis (Liu et al. 2021), suggesting that PUE decreases blood glucose through the PI3K/Akt signal pathway. It also increases glucose transporter type 4 (GLUT4) mRNA and protein levels in skeletal muscle and adipocytes (Hsu et al. 2003) and upregulates peroxisome proliferators‐activated receptor α (PPARα) expression (Wu et al. 2013), enhancing glucose uptake and improving insulin sensitivity and resistance.

In diabetic patients, dysfunction of pancreatic islet cells leads to a reduction in hepatic lipoprotein esterase activity. This decrease impairs the conversion of LDL to HDL, resulting in elevated levels of LDL in both the blood and liver, and causing lipid metabolism disorders. Additionally, abnormal secretion of adipose tissue factors exacerbates insulin resistance, further disrupting blood glucose regulation. Insulin normally regulates LDL binding and uptake through LDL receptors. In cases of insulin resistance or insufficient insulin, LDL clearance is slowed, triglyceride (TG) hydrolysis is inhibited, and blood levels of TG and LDL increase. HDL, primarily formed from lipoprotein catabolism involving TG, decreases when TG catabolism is disrupted.

Hypoglycemic tests revealed that diabetic mice had increased levels of total cholesterol (TC), TG, and LDL‐C, as well as a decrease in HDL‐C. PUE was found to improve lipid metabolism disorders caused by insulin dysfunction, regulate blood lipid levels in diabetic mice, and mitigate damage caused by type 2 diabetes mellitus. The most significant effects were observed with high‐dose PUE, aligning with findings from previous studies (Liang et al. 2019). Experiments showed that the blood lipid levels in diabetic mice are mainly regulated through interaction between the liver and adipocytes. In the liver, PUE regulates the expression of genes related to fat metabolism (e.g., acetyl CoA carboxylase, carnitine acyltransferase) and protein levels (e.g., phosphorylated AMPK, hormone‐sensitive lipase) (Zheng et al. 2015). In adipocytes, PUE activates AMPK, inhibiting the expression of the key transcription factor SREBP‐1c, thereby reducing lipid synthesis and improving dyslipidemia (Xu et al. 2021a, ).

In conclusion, PUE has been shown to have a hypoglycemic effect. Its mechanism may involve reducing FBG levels, improving glucose tolerance, increasing insulin sensitivity, and enhancing insulin resistance in diabetic mice by boosting antioxidant effects and repairing pancreatic islet cells.

A high‐cholesterol diet is one of the main risk factors for hyperlipidemia in the elderly. Long‐term consumption of a high‐fat diet results in fat accumulation in visceral organs (such as perirenal fat and epididymal adipose tissue), increasing oxidative stress and disrupting lipid metabolism, which leads to abnormal levels of total cholesterol (TC), triglycerides (TG), low‐density lipoprotein cholesterol (LDL‐C), and high‐density lipoprotein cholesterol (HDL‐C) (Ponziani et al. 2015). In this study, feeding mice a high‐fat diet was used to create a high‐fat model. Results showed that the MC group had significantly higher levels of serum TC, TG, and LDL‐C, and significantly lower levels of HDL‐C. These findings align with the study by Chu et al. (2018) showing the effects of Masson pine polysaccharide in high‐fat diet‐induced mice. Hyperlipidemia is a major risk factor for atherosclerosis, peripheral arterial disease, and other significant metabolic disorders (Watts and Karpe 2011). Elevated TC and TG levels are positively correlated with increasing risk of cardiovascular disease (Zhao et al. 2016). Therefore, lowering TC and TG levels is crucial for preventing or treating hyperlipidemia and other metabolic diseases. Studies have shown that in HFD mice, the expression levels of fatty acid synthase (FAS) and acetyl‐CoA carboxylase (ACC) in the liver are significantly increased, promoting fatty acid synthesis and leading to an increased accumulation of lipids such as TG in the liver and bloodstream. PUE significantly inhibits the gene expression of FAS and ACC, reducing fatty acid synthesis and consequently lowering TG levels in the liver and serum (Zheng et al. 2015). In the study, the levels of TC, TG, and LDL‐C in mice from both the PUE and lovastatin groups were lower versus the MC group, while the levels of HDL‐C were also increased, showing that PUE significantly lowers blood lipids and potentially improves hyperlipidemia in mice. Zheng et al. (2015) found that PUE reduces lipogenesis by downregulating the mRNA expression of peroxisome proliferator‐activated receptor γ 2 (PPARγ2), a key regulator of adipogenesis and adipocyte differentiation. Additionally, PUE upregulates the mRNA expression of carnitine acyltransferase (CAT) and hormone‐sensitive lipase (HSL), enhancing lipolysis. In summary, PUE primarily regulates the blood lipid levels in mice by modulating the expression of related genes and proteins, thereby exerting a hypolipidemic effect.

## 5 Conclusion and Limitation

This study demonstrated that PUE can effectively regulate glucose and lipid metabolism disorders associated with aging‐related metabolic diseases. By enhancing antioxidant enzyme activity, PUE exhibits both antioxidant and hypoglycemic effects, as well as hypolipidemic properties. These findings demonstrate that PUE has potential for further development as an anti‐aging drug or health food. However, this study primarily focuses on the phenotypic effects of PUE in model mice, and the specific mechanisms underlying its antioxidant, hypoglycemic, and hypolipidemic effects remain unclear, presenting certain limitations. First, the specific targets of PUE in mouse cells are yet to be identified. Future studies could use molecular docking to screen potential targets. Second, animal experiments lack an exploration of signal transduction pathways. Future research should employ western blot analysis to assess the regulatory effects of PUE on related proteins (e.g., Nrf2, HO‐1, GLUT4, PPARα) and combine this with real‐time polymerase chain reaction (RT‐qPCR) to determine the expression level of corresponding genes, thereby providing further molecular insights into the mechanism of PUE in model mice.

## Author Contributions

**Hong Ruan:** data curation (supporting), formal analysis (supporting), investigation (equal), methodology (supporting), writing – original draft (lead), writing – review and editing (equal). **Wanqing Li:** investigation (equal). **Huien Chang:** investigation (equal). **Manlin Wen:** investigation (equal). **Fangshuai Song:** investigation (equal). **Li Ye:** investigation (equal). **Jie Mei:** investigation (equal). **Shouchun Luo:** investigation (equal). **Xiqiang Zhu:** conceptualization (supporting), data curation (supporting), formal analysis (supporting), methodology (equal). **Xiaopeng Liu:** conceptualization (lead), data curation (lead), formal analysis (supporting), methodology (equal), writing – review and editing (equal). **Ning Jiang:** conceptualization (supporting), data curation (supporting), formal analysis (lead), methodology (equal), writing – review and editing (equal).

## Ethics Statement

All the experiments were conducted according to the Guidelines of Experimental Animal Administration austere published by the State Committee of Science and Technology of the People's Republic of China. The study was reviewed and approved by the Laboratory Animal Ethics Committee of Hubei Minzu University.

## Conflicts of Interest

The authors declare no conflicts of interest.

## Data Availability

The data that support the findings of this study are available on request from the corresponding authors. The data are not publicly available due to privacy or ethical restrictions.
